# Electronic Assisted Living Technology in CCRC, Assisted Living, and In-Home Care

**DOI:** 10.1093/geroni/igaa057.295

**Published:** 2020-12-16

**Authors:** John Hall, Linh Lee, Joshua Littlejohn

**Affiliations:** 1 envoyatHome, LLC, envoyatHome, Pennsylvania, United States; 2 University of Pennsylvania School of Medicine, University of Pennsylvania, Pennsylvania, United States; 3 University of Pennsylvania Health System, Philadelphia, Pennsylvania, United States

## Abstract

A qualitative study based on structured interviews with 21 healthcare leaders from CCRC’s, In-Home Care Agencies, or Medicare PACE facilities was conducted. Implications of electronic assisted living technologies on caregiver workforce were assessed. The use of assisted living technology was shown to have implications for workforce support and the alleviation of demands on caregivers. Communication and assessment tools were also found to be useful in the reduction of caregiver stress. There is optimism regarding the effectiveness of high-tech platforms in easing caregiver burden but there is skepticism about the return on investment given the initial cost and time needed for onboarding and data organization. The lack of user-friendliness and the required time to train to use tech are also barriers. The use of technology for remote check-ins and to monitor vitals is desirable. Also predicting dementia risk and monitoring for wandering are other opportunities for tech adoption. The enthusiasm for technology is tempered with caution for who will keep tabs on remote monitoring and who bears the responsibility to respond to the information gathered. Labor shortages, time constraints, and disorganized documentation are incentives for tech adoption. High-tech solutions for would ideally be user-friendly and help reduce staff demands. Except for the prevention of re-hospitalization by monitoring vitals the benefits of adopting new tech are not perceived as cost-effective.

